# Application of ferroptosis strategy to overcome tumor therapy resistance in breast and different cancer cells

**DOI:** 10.22038/IJBMS.2024.77465.16752

**Published:** 2024

**Authors:** Waniya Shahid, Ahmar Iqbal, Iram Iqbal, Arshad Mehmood, Hongyan Jia

**Affiliations:** 1 Department of General Surgery Sub Specialty Breast Surgery, Shanxi First Medical Hospital affiliated to Shanxi Medical University, Yingze District, 030000, Taiyuan, China; 2 Department of Pharmacology, Bahauddin Zakriya University Multan, Pakistan; 3 Primary & Secondary Healthcare Department, Govt. of Punjab, Pakistan; 4 Department of Neurology, The Second Hospital of Hebei Medical University, Shijiazhuang 050000, Hebei, China

**Keywords:** Drug resistance, Ferroptosis, Glutathione peroxidase, Iron metabolism, Lipid metabolism, Neoplasms

## Abstract

This literature review emphasizes the innovative role of ferroptosis in cancer treatment. Ferroptosis is a kind of deliberate cell death that is characterized by the generation of lipid peroxides and needs the presence of iron. Ferroptosis is a controlled cell death process that adheres to certain rules and regulations. The inhibition of System Xc- and the involvement of GPX4 are two of the primary areas of exploration that are engaged in the process of ferroptosis. This review explores the treatments that are used to treat ferroptosis in a range of malignancies, with a particular focus on breast carcinoma. Attention is paid to certain pathways, such as the FSP1-independent regulation of glutathione, involvement of cholesterol, and the prominin 2-MVB/exosome-ferritin pathway. Ferroptosis plays a key role in resistance to tumor therapy.

## Introduction

The term “ferroptosis” was first used in 2012 to describe a type of regulated cell death that is iron-dependent and brought on by the buildup of lipid-based reactive oxygen species. Ferroptosis is a form of regulated cell death that is characterized by the iron-dependent accumulation of lipid hydroperoxides to lethal levels. Unlike apoptosis or necrosis, ferroptosis is specifically inhibited by iron chelators and lipophilic antioxidants. This unique dependence on iron and susceptibility to lipid peroxidation distinguishes ferroptosis from other forms of regulated cell death (1). Cell death is an inevitable and crucial step in the process of life, marking the unavoidable end of a cell’s life, whether it occurs under normal or pathological circumstances (2). Based on various physical characteristics, cell death has historically been separated into three primary categories: type I cell death, also known as apoptosis; type II cell death, also known as cell death including autophagy; and type III cell death, also known as necrosis. Apoptosis was thought to be equivalent to controlled cell death (3). Only a handful of the various types of cell death that have been identified thus far include apoptosis, autophagy, necroptosis, pyroptosis, and ferroptosis. In a study, 23,550 compounds were tested via synthetic lethal high-throughput screening to see whether they could destroy lab-grown tumor cells but not their isogenic counterparts of healthy cells. They found several compounds that displayed genotype-selective effects, including leucine-rich repeat protein 5 (FXBL5) bouvardin, sangivamycin, echinomycin, mitoxantrone, camptothecin, doxorubicin, daunorubicin, mitoxantrone, camptothecin, and a new chemical we called erastin (4)**. **Ferroptosis is an identified form of programmed cell death caused by the build-up of iron-dependent peroxidation. Ferroptosis has distinctive morphological and bioenergetic properties that set it apart from other documented forms of programmed cell death (5). The program involves three basic metabolisms: iron, thiol, and lipid, resulting in cell death and iron-dependent lipid peroxidation (6). Ferroptosis demonstrates excellent possibilities in cancer therapy. Investigating the mechanics of ferroptosis has advanced significantly (7). Mice exposed to iron oxide developed lung tumors far more frequently. In a different investigation, iron excess in the liver is caused by dysregulation of the iron uptake and export systems in F-box and FXBL5 mutant mice, eventually resulting in liver tumor development (8). Cells with RAS mutations exhibit increased susceptibility to ferroptosis-inducing substances due to their elevated iron levels resulting from the mutation (9). Ferroptosis can stop the growth of cancerous cells in the liver, pancreas, breast, prostate, and other malignancies (10, 11). Inducing ferroptosis may be a novel way to treat cancer, and some highly aggressive cancer cells are intrinsically prone to it (12). These inducers include clinically approved medications and molecules in the research phase. Notably, nanotechnology presents novel opportunities for inducing ferroptosis in cancer therapy. Nanomaterials can not only compensate for the deficiencies of conventional pharmaceuticals (low aiming efficiency, poor water solubility, and serious adverse reactions) due to their unique physicochemical properties, but they can also introduce novel characteristics, e.g., magnetic property, photo-thermal effect, electrochemical property (13). Because of its non-apoptotic character, ferroptosis-based treatment is anticipated to circumvent the disadvantages of conventional therapies mediated by pathways. Recently, ferroptosis is also implicated in cancer immunotherapy, in which T cells and interferon-gamma (IFN-) sensitize tumor cells to ferroptosis (14). Following considerable investigation as a ferroptosis inducer, erastin is a small molecule drug that preferentially kills tumor cells expressing small T (sT) oncoprotein and RAS^V12 ^(15)**.**

This review summarizes the fundamental components that form the basis of the process of ferroptosis, a kind of cell death that is often resistant to cancer treatment. This explains how ferroptosis acts as a distinguishing feature of cancer treatment by controlling iron metabolism and lipid peroxidation. Ferroptosis may selectively eradicate cancer cells while preserving normal cells by focusing on these pathways, which has the potential to improve the effectiveness of other cancer treatments. Ferroptosis has the ability to regulate the tumor microenvironment, impeding tumor growth and stimulating an immune response against the tumor. Gaining a comprehensive understanding of these pathways offers useful insights into the possible uses of ferroptosis in cancer treatment, emphasizing its potential as an innovative therapeutic approach. Due to ferroptosis’s promise in cancer therapy and the fast growth of inducers in recent years, it is possible to classify and follow contemporary research. In cancer cells, targeting the cystine/glutamate antiporter system Xc- induces ferroptosis, suggesting novel therapeutic targets. New ferroptosis inhibitors have shown the involvement of lipid peroxidation and iron metabolism. This rapidly expanding area of ferroptosis research emphasizes its promise as a cancer therapy, underlining the necessity for continual monitoring and characterization. In the meantime, ferroptosis complexity and tumor resistance hinder therapy development. Yet, new inducers and research growth offer hope. Personalized treatments and biomarkers aid in overcoming challenges, potentially revolutionizing cancer therapy.

***Literature search and methodology***

In this review, different databases, including PubMed, Google Scholar, NIH National Library of Medicine, Scopus, and Web of Sciences, were surveyed to retrieve data using a series of search terms, namely “Ferroptosis, Neoplasms/therapy (for Cancer Therapy), Drug Resistance, Iron Metabolism, Glutathione Peroxidase/metabolism (for GPX4), Lipid metabolism” and articles published after the year 2000 were preferred. Research articles and reviews were included, whereas conference abstracts and non-English publications were excluded.

***Illustrations and figures***

The figures and pathway demonstrating the mechanism of action were drawn in Microsoft Powerpoint 2019, illustrator, and Biorender (https://biorender.com/, accessed on 28 February 2023).

***Ferroptosis***

Ferroptosis is a controlled cell death separate from apoptosis and necrosis, and it is vital to cancer treatment. Ferroptosis involves iron metabolism, lipid peroxidation, and erastin. Erastin lowers glutathione (GSH) and disrupts mitochondrial function to preferentially kill cancer cells with enhanced small T oncoprotein (ST) and oncogenic RAS expression (16). Recent research in the field of ferroptosis has provided specific details and examples supporting the claim that it is possible to characterize the most recent research and monitor its development. Lipid peroxides accumulate, causing cell death. Iron-chelating chemicals inhibit this cell death pattern, but RSL3 induces it (17). While RSL3 can induce it, it highlights the complex interplay of factors involved in this process.

Ferroptosis causes increased bilayer barrier density, mitochondrial cristae impairment, and mitochondrial volume reduction without cell death. GSH depletion and lipid peroxide buildup, which create ROS and cause cell death in the presence of iron, result from diminished GPX4 activity (2). The recent discovery regarding erastin’s activation of ferroptosis sheds light on its mechanism. Erastin raises the levels of protein 2a linked with lysosomes (LAMP2a), which, in turn, promotes the degradation of glutathione peroxidase 4 (GPX4) and chaperone-mediated autophagy. This process leads to an accumulation of lipid peroxides and ultimately triggers ferroptosis. Understanding these molecular mechanisms is crucial for developing targeted therapies that can selectively induce ferroptosis in cancer cells while sparing normal cells, thus paving the way for more effective and less toxic cancer treatments.

Genetic research shows that GPX4 gene deletion promotes ferroptosis, demonstrating its importance in ferroptosis control (18). The genetic regulation of ferroptosis involves several key genes that play critical roles in iron regulation and lipid peroxidation metabolism. Iron is a central player in ferroptosis, and its intracellular levels are tightly regulated. Transferrin receptor protein 1 (TFRC) is responsible for the uptake of transferrin-bound iron into cells, while ferritin heavy chain 1 (FTH1) is involved in the storage of excess iron. These genes, along with others like iron-responsive element-binding protein 2 (IREB2) and ferroportin (FPN), help maintain iron homeostasis and influence the susceptibility of cells to ferroptosis. These findings demonstrate the basic foundations of ferroptosis activation and suggest cancer treatment options targeting spontaneous cell death. Lipid peroxidation metabolism is another crucial aspect of ferroptosis regulation. Arachidonate 15-lipoxygenase (ALOX15) and acyl-CoA synthetase long-chain family member 4 (ACSL4) are enzymes involved in the synthesis and metabolism of lipid peroxides, which are key mediators of ferroptosis. These genes, along with others like lysophosphatidylcholine acyltransferase 3 (LPCAT3) and glutathione peroxidase 4 (GPX4), play critical roles in modulating lipid peroxidation levels and influencing cell death pathways.

The complexity of the living process and the difficulty of clinical translation present both obstacles and opportunities for the advancement of ferroptosis-based treatment of cancer. For example, one challenge is the need to develop targeted therapies that selectively induce ferroptosis in cancer cells while sparing normal cells. Additionally, the heterogeneity of cancer cells and the complexity of signaling pathways involved in ferroptosis regulation pose challenges to developing effective treatments. However, advances in understanding the molecular mechanisms of ferroptosis and the development of novel therapeutic agents offer opportunities for the development of more targeted and effective cancer treatments based on ferroptosis.

***Regulatory mechanisms of ferroptosis***

**Figure 1:**
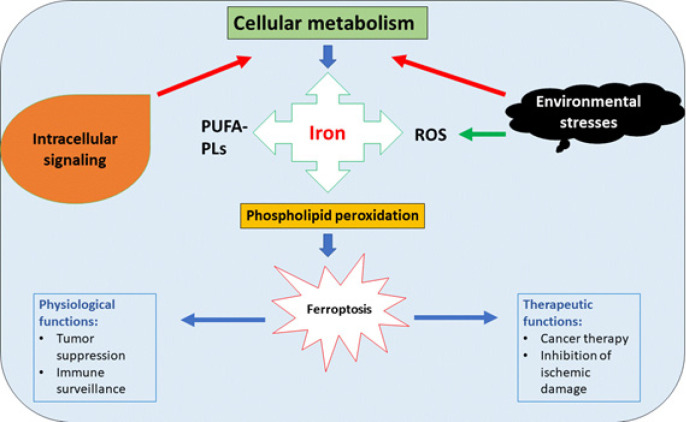
A schematic diagram illustrating the process of ferroptosis

Ferroptosis is known to be mediated by iron metabolism and lipid peroxidation signaling (19). Ferroptosis meets this RCD requirement because it may be stopped by reducing lipid peroxidation or depleting iron pharmacologically or genetically. Due to cell metabolism and unstable redox equilibrium, lipid peroxidation causes lethal oxidative damage (20). Ferroptosis may affect immune function and tumor suppression, according to accumulating studies. Ferroptosis may be prevented by directly decreasing lipid peroxidation or lowering iron, which may treat cancer. It may be inhibited by targeting lipoxygenases (LOX) or iron chelators such as deferoxamine (DFO) or deferiprone (19). Ferrostatins and liproxstatins are promising lipid peroxidation inhibitors in preclinical research (20). Genetic techniques that target iron metabolism or lipid peroxidation genes may also prevent ferroptosis. Directly suppressing lipid peroxidation or lowering iron may prevent ferroptosis and lead to new cancer treatments. Ferroptosis processes and treatment targets are presented in Figure 1.

***Iron metabolism pathway***

**Figure 2:**
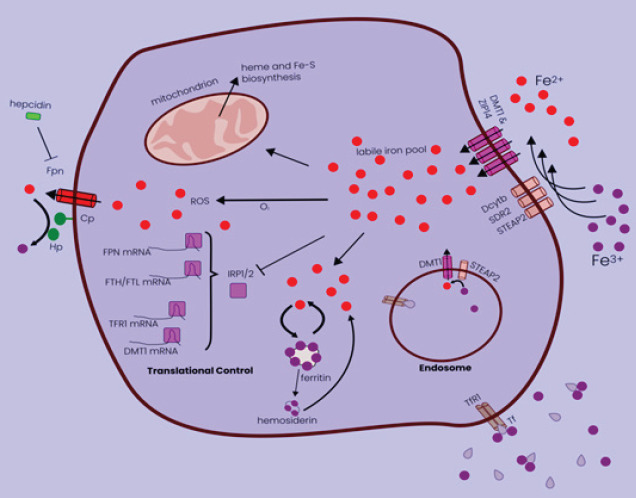
Diagram displaying a broad perspective on human cellular iron homeostasis

The body needs iron, which is an essential trace element. The body’s abnormal iron concentration and distribution can have an impact on typical physiological functions. Despite it having “iron” in the title, the role of iron in ferroptosis and how it is controlled are relatively new ideas. The lipid peroxidation that causes ferroptosis is caused by iron-dependent processes alone or if the unstable iron pool interacts with lipid peroxides to distribute these species in membranes (21). Ceruloplasmin can oxidize Fe2+ produced by digestion or erythrocyte breakdown to Fe3+ (22). The main protein in charge of transporting iron is called transferrin (23). Intracellular labile iron (redox-active Fe2+) level can be increased by TF-mediated iron absorption or autophagic/lysosomal ferritin degradation, and it is essential for triggering oxidative damage and ferroptosis (ferritinophagy)(24). The build-up of iron is the initial stage of ferroptosis. A significant amount of extracellular transferrin (Tf) and free iron (Fe3+) in the blood combine to create a complex (TF-Fe3+). Tf then binds to the membrane protein transferrin receptor 1 (TFR1) on the cell membrane, allowing for endocytosis to take place and transporting iron into the cell (25). The six transmembrane epithelial antigens of prostate 3 (STEAP3) then cause the degradation of Fe3+ to the highly reactive Fe2+. Fe2+ moves from endosomes to the cytoplasm by the transporter of divalent metals 1 (DMT1), where it generates an unsteady iron pool. A portion of the iron pool’s Fe2+ is kept in ferritin to shield cells and tissues from harm caused by iron, while a second portion of the Fe2+ can be expelled from cells across the cell membrane’s ferroportin (FPN)(26). Cellular iron homeostasis is rigorously regulated by this internal iron recycling (Figure 2). Under usual conditions, intracellular iron concentrations are constant. The right amount of iron keeps the body’s physiological processes operating normally, while too much free iron can damage cells (27). When the equilibrium is upset, like when the iron is overloaded, too much Fe2+ is formed inside the cell, which allows the Fenton chemical reaction to catalyze the production of Fe3+ and hydroxyl radicals directly. The most dangerous oxygen free radicals in the human body are hydroxyl radicals, which are also extremely potent ROS. Oxidation of lipids and ferroptosis are the results of their easily obtaining electrons from other molecules. The superoxide radical (O2) reaction, sometimes referred to as the Haber-Weiss reaction, may decrease Fe3+ back to Fe2+ (28). Numerous recent research have traced the direct relationship between iron and the incidence of ferroptosis, and they show that elastin-induced ferroptosis may be inhibited by suppressing the expression of genes (TFRC) regulating TFR-1 (29). However, elastin-induced ferroptosis is accelerated by heme oxygenase-1 (HO-1) synthesis by increasing iron levels (30). The heat shock protein beta-1 (HSPB1), which is overexpressed, dramatically inhibits ferroptosis and can further lower intracellular iron concentrations by suppressing TRF1 production (31).

***Regulation of the lipid metabolism pathway***

**Figure 3:**
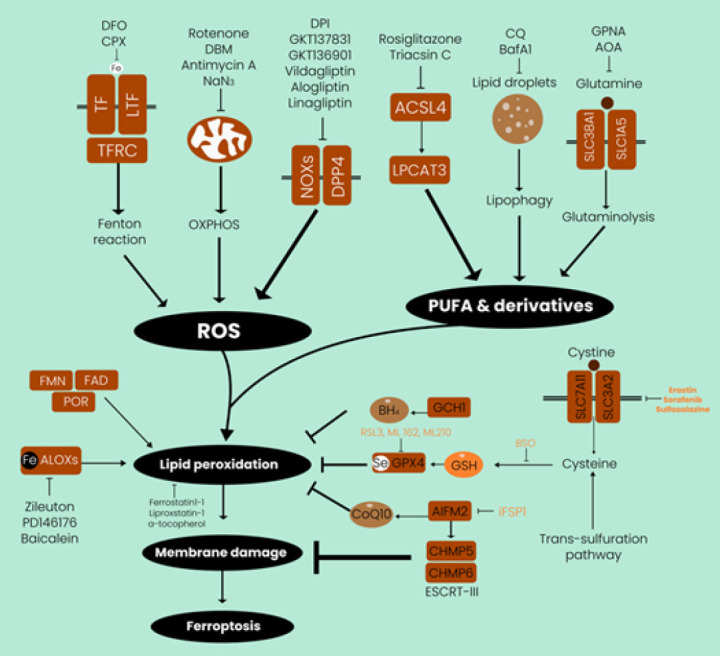
Ferroptosis is a form of cell death that occurs due to lipid peroxidation of polyunsaturated fatty acids (PUFAs)

Ferroptosis, which kills cells, involves lipid peroxidation, oxidative damage, and abnormal redox equilibrium. Ferroptosis relies on lipid peroxidation, oxidative breakdown of cell membrane lipids, and PUFAs. Lipid peroxides impair cell membrane structure and function. This disturbance may produce membrane holes and barrier function loss, affecting membrane binding and thickness. The alterations reduce cell viability and cause cell death (32). Oxidative damage occurs when lipid peroxides and other ROS from lipid peroxidation react with proteins, lipids, and DNA/RNA. Damage to cellular equilibrium may cause ferroptosis and other cell death mechanisms (33). Ferroptosis is caused by ROS such as H2O2, O2-, 1O2, and other free radicals that damage biomolecules and kill cells. Ferroptosis promotes lipid peroxidation and oxidative damage due to an imbalanced redox equilibrium with increased ROS and oxidative stress. Oxidative compounds, such as ferroptosis-indicating phospholipid hydroperoxides, may accumulate when redox equilibrium is disrupted. This imbalance increases lipid peroxidation and cell death (34). Lipid peroxidation, oxidative damage, and disturbed redox equilibrium in ferroptosis provide the relevance of lipid homeostasis and redox balance for cell survival. Ferroptosis may cause cell death if these mechanisms are disrupted, which has implications for cancer research and treatment (Figure 3).

***Role of mitochondria in ferroptosis***

**Figure 4:**
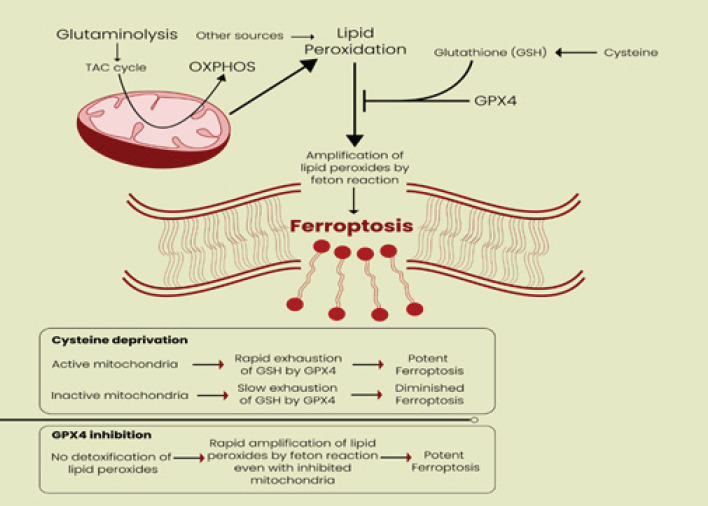
Mitochondria play a crucial role in ferroptosis

Contrary to common opinion, mitochondria are crucial to the apoptotic process. Mitochondrial lipid membrane permeabilization (MOMP), which occurs when mitochondrial apoptosis is initiated, usually results in cell death. Ferroptosis is characterized by a condensed mitochondrial transmembrane density, a decreased mitochondrial volume, a diminished or absent mitochondrial crista, and damaged cellular membranes (35). Mitochondria play a key role in cysteine-deprivation-induced ferroptosis but not in that induced by inhibiting glutathione peroxidase-4 (GPX4), the most downstream component of the ferroptosis pathway (36). Mechanistically, cysteine deprivation leads to mitochondrial membrane potential hyperpolarization and lipid peroxide accumulation (Figure 4). Transmembrane channels, known as VDACs, carry ions and metabolites and are crucial regulators of ferroptosis (37). According to Yagoda *et al*., erastin operates on VDACs to cause mitochondrial malfunction, a huge quantity of released oxides, and iron-mediated cell death (38). Macrophages also undergo ferroptosis (39, 40). The potential of the mitochondrial membrane is hyperpolarized, and a build-up of lipid peroxide and ferroptosis is all reduced by inhibition of the mitochondrial TCA cycle or electron transfer chain (ETC). The similar inhibitory impact of blocking glutaminolysis is reversed by providing downstream TCA cycle intermediates. The tumor suppressor and TCA cycle component fumarate hydratase’s loss of function is significant because it provides resistance to cysteine-deprivation-induced ferroptosis. Together, these findings suggest how important mitochondria are in ferroptosis caused by cysteine deficiency and suggest that ferroptosis has a role in tumor suppression (36, 41).

***Mechanisms inducing ferroptosis***

Ferroptosis is identified during a chemical screening for cancer treatment, revealing various compounds that cause cell death. These substances promote ferroptosis through different methods. Glutamate, sulfasalazine, and erastin induce ferroptosis by inhibiting the cystine-glutamate transporter (Table 1). This depletion of cysteine and glutathione increases lipid peroxidation and cell death (42). On the other hand, RSL3 and DPI7 induce ferroptosis by inhibiting glutathione peroxidase 4 (GPX4) activity. GPX4 protects cells from lipid peroxidation by converting lipid hydroperoxides to alcohols (43). Identifying novel ferroptosis-inducing compounds impacts cancer therapy, as understanding how these substances promote ferroptosis can lead to targeted medicines that kill cancer cells without harming normal cells. Cancers reliant on cystine-glutamate transporters or GPX4 may benefit from targeting these pathways. Understanding ferroptosis induction mechanisms is crucial for creating new cancer treatments.

***Provoking ferroptosis by system Xc-suppression***

In the phospholipid bilayers system, Xc-, which is an antitransporter for amino acids, is found in large quantities. It is a heterodimer made up of the two subunits, *SLC7A11* and *SLC3A2*, and it is a component of a crucial antioxidant mechanism in cells. System Xc- exchanges glutamate and cystine in a 1:1 ratio into and out of the cell (6). Cells convert the cysteine that is taken into cysteine, which is used to create GSH. Glutathione peroxidases function to decrease by way of GSH, ROS, and oxidative nitrogen, which are reduced (GPXs). By preventing the absorption of cystine, the activity of system Xc- impacts the production of GSH, GPX activity, and cellular antioxidant capability. This affects, among other things, the build-up of lipid ROS and the eventual development of oxidative stress and ferroptosis (44, 45).

***Role of GPX4 in ferroptosis induction***

Ongoing research is being done to create cancer therapeutics based on ferroptosis induction. While several untargeted nanocarrier approaches for delivering iron, oxidizing agents, and other harmful payloads to damage cancer cells have been explored, the existence of numerous enzymes that regulate ferroptosis makes it possible to create targeted methods. GPX4, which is essential for cancer cell survival and is expressed in the majority of cancer cell lines, is possibly the most obvious target (46). A multifunctional protein called GPx4 may reduce peroxidized lipids in their free form in combination with other proteins or lipids like lipoproteins or PLs or even within membranes (47). Lipid hydroperoxides are converted to lipid alcohols by the lipid hydroperoxidase glutathione peroxidase 4 (GPX4), preventing the iron (Fe2+)-dependent production of harmful lipid reactive oxygen species (ROS) (48). A sign of ferroptosis is the build-up of lipid peroxides, which can happen when GPX4 function is inhibited. According to Yang *et al*., cells with reduced GPX4 expression are more susceptible to ferroptosis, in contrast to cells with increased GPX4 expression, which stops ferroptosis (49). RSL3, a ferroptosis inducer, directly interacts with GPX4 and suppresses its activity, lowering cells’ ability to fight off ROS and causing ferroptosis (50). Among the necessary amino acids for the active group of GPX4 is selenocysteine (50). Due to the fact that covalent GPX4 blockers alter GPX4’s selenocysteine terminus and that of extra selenoproteins, GPX4 missing a traditional tiny molecule attaching site selectivity is a concern. These inhibitors are also very unstable since they are extremely reactive. However, by developing disguised prodrugs that can be transformed metabolically into their stable metabolite inside cells, this issue can be resolved. However, the primary caution still stands that because GPX4 is required for many tissues in the periphery, Targeting GPX4 in mouse kidney tubular cells and certain neuronal subpopulations is anticipated to have considerable negative effects (51). Another strategy is to directly block GPX4 since the presence of selenocysteine tRNA is needed to incorporate selenocysteine in GPX4; a group of isopentenyl lipids, the mevalonate (MVA) pathway’s byproduct, must first be added to the Sec-tRNA in order to activate it (52, 53). This may help to explain why statins’ interference with the MVA route causes certain cells to express less GPx4 and undergo more ferroptosis (11).

***Ferroptosis regulation in different cancers***

Ferroptosis has become a prominent phenomenon in cancer research, with initial studies mostly aimed at discovering chemical agents that might cause ferroptosis as possible treatments for cancer. Ferroptosis is a kind of cell death that relies on iron and is distinct from apoptosis and autophagy. It is characterized by the build-up of ROS inside the cell. Cancer cells, because of their heightened metabolic activity and increased accumulation of ROS, are thought to be more vulnerable to ferroptosis. Research has shown that cancer cells often display disrupted regulation of iron levels and increased reliance on iron, a condition referred to as iron addiction, which may stimulate the development of tumors. By specifically addressing the dysregulation of iron metabolism and activating the pathways that lead to ferroptosis, it may be feasible to overcome inherent drug resistance mechanisms in cancer cells, providing a novel approach to cancer treatment. Several forms of cancer, including lung cancer, kidney cancer, ovarian cancer, and breast cancer display abnormal regulation of iron levels, which further supports the involvement of ferroptosis in the advancement of cancer. In the case of lung cancer, the abnormal regulation of iron metabolism is associated with the growth and survival of tumor cells (65). Similarly, elevated iron levels are linked to the aggressive nature of tumors in breast cancer (66). Iron-dependent cell senescence, a process in which cells enter a state of irreversible growth arrest due to excessive iron levels, has been implicated in promoting ferroptosis in cancer cells. Targeting dysregulated iron metabolism through approaches like iron chelation or inhibiting iron-related proteins could induce ferroptosis and inhibit tumor growth, offering a potential therapeutic strategy for cancer treatment. Gaining insight into the precise processes by which ferroptosis is controlled in various types of cancer is essential for the development of focused, therapeutic approaches. A summary of different published research regarding the ferroptosis sensitivity of different types of malignant cells is presented (Table 2).

***Ferroptosis and breast carcinoma***

A large portion of female breast cancer deaths are due to triple-negative breast cancer (TNBC), which accounts for 15-18% of cases. TNBC prognosis is poor because of restricted targeted treatments, generally chemotherapy. (75). TNBC patients had higher mucin 1 (MUC1-C) levels. Cysteine is very important when interacting with system Xc-’sIn TNBC. Ferroptosis, characterized by iron-dependent lipid peroxide build-up, is caused by decreased system Xc- activity and cystine consumption xCT light chain (MUC1-C) maintains Glutathione (GSH) levels and redox equilibrium. Together with CD44v, this interaction modulates GSH levels. MUC1-C/xCT pathway inhibition causes ferroptosis in TNBC cells, reducing self-renewal (76). Inducing ferroptosis using Erastin, RSL3, ML210, and ML162 reduces breast cancer cell efficacy. Specific ferroptotic drugs like Erastin, RSL3, ML210, and ML162 are less effective against breast cancer cells. This is because although these drugs are capable of inducing ferroptosis, their efficacy in reducing breast cancer cell viability is limited. This limitation could be due to several factors, such as the heterogeneity of breast cancer cells or the presence of mechanisms that counteract the effects of these drugs. Silamesine, a lysosome disruptor, and lapatinib, a tyrosine kinase inhibitor, induce ferroptosis, according to studies. Silamesine and lapatinib increase transferrin expression and decrease ferroportin action to increase cellular iron. These substances kill cells, while ferroptosis inhibitors prevent it (67). A Fe2+-based metal-organic skeleton provides Fe2+ to cancer cells, causing the Fenton reaction and excessive ROS production, producing ferroptosis in breast cancer cells. Molecular markers related to ferroptosis may predict breast cancer patients’ overall survival (OS). From The Cancer Genome Atlas (TCGA) ferroptosis-linked Differentially Expressed Genes (DEGs), a predictive multigene signature is created. Gene Expression Omnibus (GEO) confirmed these signatures. To understand ferroptosis in this setting, lncRNAs, miRNAs, and the immune response in breast cancer must be considered (77).

***Ferroptosis and drug resistance***

SH/GPX4 controls ferroptosis-induced inflammation. These concerns need further investigation. Ferroptosis, like apoptosis (caspase activation) and autophagy, has no specific indications. Thus, ferroptosis indicators must be studied. As research progresses, ferroptosis-regulating mechanisms may be novel. Ferroptosis is found in the pathophysiological pathways of several disorders, suggesting a potential treatment (78). Ferroptosis, a cell-killing method, may also contribute to illnesses in combination with other methods, allowing the joint use of current treatment plans and helping to resolve drug resistance issues in certain diseases. Many apoptotic factors rely on activating P53 to regulate apoptosis. Ferroptosis is also controlled by P53. In certain cases, P53 may prevent ferroptosis via the P53-P21 axis, and system Xc- down-regulates *SLC7A11* expression (79). Autophagy contributes to ferroptosis. Ferritin changes during autophagy. In the ATG5-ATG7-NCOA4 pathway, autophagy coupled with ferritin may increase ferroptosis by increasing unstable iron in cells. Thus, autophagy, apoptosis, and ferroptosis control are comparable. Ferroptosis might worsen due to iron ion alterations. Free intracellular Fe2+ may create hydroxyl or peroxide radicals, harming lipids via the Fenton reaction. Copper, a distinct transition metal, affects ferroptosis and glutamate-induced oxidation in HT22 cells and is linked to redox metabolism. Thus, ferroptosis may be influenced by metal ions other than iron. The original ferroptosis idea is substantially undermined; deeper examination is needed. When upstream iron metabolism genes like FPN, TFR1, and DMT1 affect ferroptosis, the downstream pathway changes. There is accumulating evidence that ferroptosis may cause inflammation and activate the innate immune system via cell components, which influences inflammatory damage, signal transmission, and cell formation. Research suggests GPX4 activation might be used to develop a new cytoprotective or anti-inflammatory medication (9).

***Ferroptosis resistance caused by a suppressor FSP1 independent of glutathione***

Despite metabolic limitations and phospholipid composition being crucial variables, no cell-autonomous mechanisms have been found to explain cell resistance to ferroptosis. An expression cloning approach can identify human tumor cell genes that replace GPX4. Recently, the flavoprotein apoptosis-inducing factor mitochondria-associated 2 (AIFM2) was shown to be a powerful antiferroptotic gene involved in mitochondria synthesis. Apoptosis Suppressor Protein 1 (FSP1), formerly AIFM2, protects against ferroptosis when GPX4 is deleted. This gene is ferroptosis suppressor protein 1. Ubiquinone (CoQ10) reduces ferroptosis via FSP1(80). Novel cholesterol-metabolizing compounds such as SOAT1, SQLE, and NPC1 are promising cancer treatments (81). Although GPX4 glutathione peroxidase 4 and radical-restricting antioxidants are thought to be the key modulators of ferroptosis, additional aspects should also be considered.

***Role of cholesterol in ferroptosis resistance***

Normal human cells can either produce cholesterol on their own or absorb it from lipoproteins to suit their metabolic needs. In some cancerous cells, *de novo* cholesterol production genes may be transcriptionally suppressed or changed, which necessitates the absorption of cholesterol from lipoproteins for the cells to survive. According to recent research, lymphoma cells that rely on lipoproteins to transport cholesterol are also susceptible to ferroptosis, an oxygen- and iron-dependent cell death process induced by a buildup of oxidized lipids in cell membranes, unless the lipid hydroperoxidase glutathione peroxidase 4 (GPX4) lowers these hazardous lipid species (82). Cholesterol is among the key elements affecting ferroptosis by causing resistance. This process can be caused by multiple factors. Studies show that severe exposition to 27-hydroxycholesterol exposure in healthy cells, a cholesterol metabolite circulating abundantly, targets the cells with more capacity. These types of cells are more vulnerable to tumor generation. For these cells to sustain a proper function in the presence of excess lipids, they need a balanced presence of GpX4, a stimulator, to prevent resistance. Resistance to ferroptosis can be an important marker for the growth of tumors and other cancers, which can be stopped with the expression of GPX4, which helps to knock down the metastasis in cancer cell lines 27HC or any other tumor growth (83). Ferroptosis resistance can be a valid reason for the genesis of dyslipidemia/hypercholesterolemia in a cell that can further cause lipid oxidation and further affect cancer pathogenesis. Dyslipidemia and hypercholesterolemia are linked to worse outcomes in people with advanced illness, as well as an elevated risk for many different forms of cancer. We find that persistent exposure of cells to 27-hydroxycholesterol (27HC), a common circulating cholesterol metabolite, favors cells with higher cellular uptake and/or lipid production. The exact processes by which this happens are complex. These cells have a significantly higher capability for tumorigenesis and metastasis. Interestingly, the metabolic stress caused by the accumulating lipids forces cells to produce GPX4, a regulator of ferroptotic cell death. In addition to demonstrating that GPX4 knockdown reduces the heightened tumorigenic and metastatic activity of 27HC-resistant cells, we also demonstrate that ferroptosis resistance is a characteristic of metastatic cells (83). High levels of biological activity can be seen in the byproducts of lipid peroxidation chain reactions. It also serves as a molecular switch to turn on signaling pathways that start cell death because of destroying DNA, proteins, and enzyme function**. **The autophagy and membrane repair mechanism work in coordination with several oxidative and antioxidant systems to modify the lipid peroxidation process throughout ferroptosis (84).

***Prominin 2-MVB/exosome-ferritin pathway-mediated ferroptosis resistance***

Prominin 2, a pentaspanin supermolecule involved in controlling lipid dynamics, is expressed as a result of ferroptosis-promoting stimuli, inhibition of the macromolecule hydroperoxidase GPX4, and dissociation from the living thing matrix. In breast cancer cells and ductal glands of animal tissue, prominin 2 improves ferroptosis resistance. Our findings suggest that the prominin 2-MVB/exosome-ferritin pathway may be responsible for ferroptosis resistance and have extensive ramifications for iron homeostasis, cellular trafficking, and cancer (85).

***Chemotherapy and drug resistance***

Drug resistance has proven difficult to overcome in anticancer treatment and is a significant barrier to patients’ long-term survival (86). Cancer cells frequently figure out how to become resistant to chemotherapy. The two basic categories of drug resistance in cancer are intrinsic (primary) and developed (secondary) resistance. Common causes of intrinsic drug resistance include aberrant cell state, genetic alterations in the tumor, and the hasty chemotherapeutic adaptation of cancer cells. The lack of an objective clinical response to therapy is one way that intrinsic medication resistance is manifested. After an initial clinical response, cancer may return locally or distantly. This is known as acquired treatment resistance (87). The poor first response to treatment is frequently the result of tumors, such as melanoma, renal cell carcinoma, and hepatocellular carcinoma that are intrinsically resistant to chemotherapy and have never undergone anticancer medications before. Tumors that have acquired drug resistance initially respond well to chemotherapy and exhibit good therapeutic responses, but later therapies fail to work as well, leading to disastrous outcomes (88). Decreased drug activity or rapid drug inactivation, excessive drug export or reduced drug absorption and delivery, delay in apoptosis, or resistance to ferroptosis are some examples of drug-related side effects that are some of the molecular mechanisms behind chemotherapy resistance. Commonly used chemotherapeutic drugs are divided into five categories based on how they work: alkylating agents, antimetabolites, topoisomerase inhibitors, mitotic spindle inhibitors, and miscellaneous (89). Chemotherapeutic substances may be made synthetically or from plants. Topotecan and sirinotecan are examples of topoisomerase I inhibitors. Teniposide, etoposide, and anthracyclines are instances of topoisomerase II inhibitors (andidarubicin, daunorubicin, and doxorubicin [DOX]). These medications prevent topoisomerases from doing their jobs, which results in DNA replication and double-strand breaks (90). Alkylating drugs include those based on platinum (ifosfamide and cyclophosphamide), oxazsaphosphorines (cisplatin, oxaliplatin, and carboplatin), nitrogen mustards (chlorambucil, busulfan, and melphalan), and hydrazine (temozolomide). Antimetabolites include ribonucleotide reductase inhibitors (hydroxyurea), purine analogs (azathioprine, 6-mercaptopurine, and cladribine), 5-fluorouracil, with cytarabine (5-FU), capecitabine, gemcitabine, and antifolates are purine antagonists (pemetrexed, methotrexate, and pralatrexate). Mitotic spindle inhibitors include Vinca alkaloids and taxanes (paclitaxel and docetaxel) (vincristine and vinblastine) (89). Over the past 30 years, therapeutic medicines for inducing apoptosis have been thoroughly explored; however, peroxidation and resultant membrane damage remain the primary causes (6). The iron build-up is the primary cause of ferroptosis, which results in the peroxidation of lipids and disruption of the cell membrane. Either intrinsic or extrinsic mechanisms can cause ferroptosis. Interfering with the expression or function of intracellular antioxidant enzymes, such as GPX4, causes the intrinsic route to become active (GPX4). Inhibiting the extrinsic route is initiated by activating the iron carriers transferrin and lactotransferrin or the amino acid antiporter system Xc (91). The two components that comprise system Xc are connected by a disulfide bond: *SLC7A11*, also known as xCT, and *SLC3A2*, also known as CD98 or 4F2hc, are light chain subunits. Two essential signals, a buildup of free iron and suppression of the antioxidant *SLC7A11*-GSH-GPX4 system, are necessary for the onset of ferroptosis (84). Due to their innate peroxide susceptibility, PL-PUFAs serve as lipid peroxidation (LPO) substrates in ferroptosis. By activating and incorporating free PUFAs into phospholipids, enzymes such as acyl-coenzyme A [CoA] synthetase long-chain family member 4 (ACSL4) and lysophosphatidylcholine acyltransferase (LPCATs) produce PL-PUFAs. In addition to dietary and environmental sources, acetyl-CoA carboxylase can be used to synthesize PUFAs from acetyl-CoA, the basic building block (ACC) (92). Once PL-PUFAs have been incorporated into the plasma membrane, iron-dependent enzymes, such as lipoxygenases and cytochrome P450 oxidoreductase (POR), are activated, and labile iron engages in a peroxidation reaction with molecular oxygen (O2) to produce PL-PUFA-OOH (92). In order to carry out this activity, either hydrogen peroxide (H2O2) must be produced by the iron-dependent Fenton reaction or POR, NADPH oxidase (NOX), or the mitochondrial electron transport chain must be activated. Labile iron can be imported because of transferrin receptor 1 (TfR1), which is then stored as ferritin and destroyed through ferritinophagy (93). A kind of nuclear receptor activator 4 (NCOA4)-mediated autophagy known as ferritinophagy degrades ferritin, creating a feedback mechanism for controlling the amount of iron that is available to cells. When this mechanism is activated, the amount of iron that is available to cells increases. In order to facilitate the movement of subcellular ferritin to autophagy lysosomes and liberate free iron, ferritin’s ferritin heavy chain-1 (FTH1) interacts with NCOA4 as a selective autophagy receptor. By controlling the homeostasis of iron and generating ROS in cells, ferritinophagy contributes to the induction of ferroptosis (94). LPO or its byproducts, such as MDA and 4-HNE, are the final stage of ferroptosis and cause pore development in the endosome or cell membranes, which results in cell death, either directly or indirectly. Ferroptosis has received a lot of interest from the cancer research community. New therapeutic approaches to get beyond cancer’s chemotherapeutic drug resistance may be found to induce ferroptosis (95-97).

We first summarize the fundamentals of chemotherapy resistance in cancer therapy and then quickly the fundamentals of ferroptosis in cancer resistance to antibiotics. We sum up the mechanism of overcoming chemotherapy resistance by inducing ferroptosis pharmacologically from studies on several cancer types. This review suggests the therapeutic possibility of pharmacologically inducing ferroptosis by bioactive substances as a means of combating chemotherapy resistance.

**Table 1:** Ferroptosis inducing agents

Reagents/compound	Target	Mechanism	Ref**e**rences
Cisplatin	GSH	GSH levels are reduced and GPXs are inactivated	(54)
Statins	HMG	CoQ10 deletion	(55)
RSL3	GPX4	Down-regulation of GPX4 and elimination of GSH	(19, 50)
BSO, DP12	GHS	GHS elimination	(50)
Sulphasalazine	System xc−	Cystine deprivation	(56)
Sorafenib	System xc−	Cystine deprivation	(57)
FIN56	CoQ10 & GPX4	CoQ10 elimination and GPX4 down-regulation	(58)
Artemisinins	Iron-related genes	Surged intracellular iron levels	(59)
DPI, ML162 compounds	GPX4	GPX4 down-regulation, as well as GSH elimination	(60)
Erastin and its derivatives	System Xc−; VDAC2/3	Cystine Loss	(19)
FINO2	GPX4	GPX4 down-regulation and lipid peroxide accumulation	(61)
BAY 87-2243	Mitochondrial respiratory Chain	Mitochondrial respiratory chain restriction (CI)	(62)
Siramesine, lapatinib	Ferroportin, Transferrin	A surge of intracellular iron	(63)
Trigonelline, brusatol	Nrf2	Nrf2 Inhibition	(64)

**Table 2:** Ferroptosis-sensitive cancer cells

Cancer cells	Ferroptosis agents	Types of evidence	References
Breast cancer cells	Erastin, Siramesine, Lipatinib	Cell culture, Tumor xenograft model	(67)
Renal carcinoma cells	BSO, Sorafenib, Erastin, RSL3	Cell culture, Tissues from patients, and a mouse model	(50)
Ovarian cancer	Erastin	Cell culture, Tumor xenograft model, Tissues from patients	(68, 69)
Glioma tumor cells	RSL3, Sulfasalazine, Erastin	Cell cultivation	(70)
Acute lymphoblastic leukemia	RSL3	Cell cultivation	(71)
Human cervical carcinoma cells	Erastin	Cell cultivation	(31)
Human hepatocellular carcinoma	Erastin, Sorafenib, DPI compounds, trigonelline, brusatole	Cell cultivation,Tumor xenograft model	(72)
Pancreatic carcinoma cells	Artesunate, Sorafenib, Erastin	Cell cultivation	(59)
Non-small cell lung cancer in humans	Erastin, Sorafenib, RSL3, M162	Cell culture	(54)
Diffuse Large Bcell lymphomas	RSL3, Sulfasalazine, Erastin	Cell culture	(50)
Colorectal cancer	Erastin, Cisplatin	A tumor xenograft model and cell culture	
Myeloid leukemia in adults	Erastin	Cell cultivation	(73)
Rhabdomyosarcoma cells	RSL3, Erastin	Cell culture	(74)

Phospholipid peroxidation relies on the metabolic byproducts reactive oxygen species (ROS), polyunsaturated fatty acid chains (PUFA-PL), and transition metal iron and indicates signaling events within and between cells, as well as environmental factors, can affect ferroptosis by avoiding the regulation of cellular metabolism and ROS levels. Ferroptosis affects health and provides some possible physiological uses

Both the ferrous iron importers DMT1 and ZIP14, which depend on the activities of iron reductases, such as STEAP2, SDR-2, and Dcytb, and endocytosis of transferrin receptor 1 can import iron. By iron-responsive element-binding proteins (IRP1/2), intracellular iron can be stored in ferritin and used for protein production or to produce reactive oxygen species (ROS) and control transcription. Hephaestin (Hp) and/or ceruloplasmin (Cp) frequently assist ferroportin in export, which is inhibited by hepcidin

The process is triggered by the production of reactive oxygen species (ROS) from various sources, including mitochondria and plasma membrane NOXs. The ACSL4-LPCAT3 pathway, lipophagy, and glutaminolysis activate ferroptosis by supplying PUFAs and related derivatives. Increased activity of enzymes involved in lipid peroxidation, such as ALOXs or POR, also contribute. Antioxidant systems like the system xc-GSH-GPX4, GCH1-BH4, and AIFM2-CoQ10 can be activated to reduce lipid peroxidation and stop ferroptosis. By a membrane restoration mechanism controlled with ESCRT-III building, AIFM2 can also prevent ferroptosis. There are several ferroptosis inducers and inhibitors

A form of cell death triggered by lipid peroxidation of polyunsaturated fatty acids. In cystine deprivation and GPX4 inhibition, the principal location of ROS generation is due to mitochondria, which promote lipid peroxidation and subsequent ferroptosis. Mitochondria also regulate iron metabolism, which is involved in ferroptosis

## Conclusion

To sum up, this review emphasizes the growing importance of ferroptosis as a potential solution to combat resistance to cancer treatment by exploring the complex mechanisms of ferroptosis and its connections with conventional treatment methods like chemotherapy and immunotherapy. Ferroptosis has the potential to revolutionize cancer treatment by targeting treatment-resistant tumors while preserving healthy tissues. As we delve deeper into the intricacies of ferroptosis and apply these discoveries to real-world applications, we are on the brink of transforming cancer treatment approaches and enhancing patient results. Exploring the potential of ferroptosis could revolutionize the field of oncology and pave the way for more precise medical treatments. 

## Data Availability

All processed data used in this review can be obtained from the corresponding author upon reasonable request.
